# The Early Diagnosis of General Paralysis of the Insane

**Published:** 1909-08-21

**Authors:** Charles Mercier

**Affiliations:** Physician for Mental Diseases, Charing Cross Hospital, and Lecturer on Insanity in the Medical School


### THE EARLY DIAGNOSIS OF GENERAL PARALYSIS OF THE INSANE.

By CHARLES MERCIER, M.D., F.R.C.P., F.R.C.S.; Physician for Mental Diseases, Charing Cross Hospital, and Lecturer on Insanity in the Medical School.

(Abstract of a Lecture delivered at the Polyclinic.)

The diagnosis of general paralysis of the insane in its early stage is often a matter of great difficulty. I speak of the pre-paralytic stage. When once the physical symptoms are present there is rarely much difficulty. It is often taken for granted that general paralysis means grandiose delusion, and grandiose delusion means general paralysis. This is not the case; general paralysis may exist and be pronounced and manifest without grandiose delusion, and without any delusion; and grandiose delusion of very exaggerated type may exist quite apart from general paralysis.

The first thing to do when general paralysis is suspected is to examine the pupils, and if the pupillary signs are present, general paralysis may be diagnosed almost with certainty; but these signs are often not found because they are not looked for, and they are not looked for because the suspicious signs, which should send us to look for them, do not arouse suspicion. They pass unnoticed, or at any rate they do not suggest a search for the physical signs of general paralysis. It is important, therefore, to recognise the symptoms that should arouse suspicion; and it is the more important because general paralysis is a progressive disease with a fatal termination, while the diseases for which it may be mistaken are for the most part recoverable.

First and most frequent among the early signs of general paralysis are the moral changes—a moral deterioration. The truthful man becomes a liar, the modest man brags, the sober man takes to drink, the chaste man consorts with loose women. When this change takes place in a man between 40 and 50, we should at once think it may perhaps be general paralysis. Usually it is put down to drink, and often the man has taken for the first time in his life to drink too much. But his drinking is an additional sign of his general paralysis, not the cause of his moral deterioration. Most characteristic among the moral changes is a lack of reticence. He talks freely of his private affairs before strangers, in railway carriages, and public places; he brags of his success in business and laments his family troubles. He laughs inappropriately, or perhaps weeps, in public.

There may be mental changes other than moral, the most frequent of which is forgetfulness; and the lapse of memory seen in general paralysis is quite distinguishable from that of old age and from that of alcoholism. In the latter cases the patient forgets events that have recently happened. He does not remember interviews that he has had, letters that he has written, business that he has transacted, even on the same day. In general paralysis what he forgets is not so much what he has done, as what he has to do. He forgets to keep appointments, to write letters, to transact business. Then he complains that his mind is confused, that he cannot concentrate his thoughts, that there is a loss of grip and inability to think clearly. These mental failures should lead us to think of general paralysis, and to investigate further.

Almost any form of transient paralysis in a man of the right age should arouse the same suspicion. Ptosis, strabismus, dragging of a limb, giddiness, aphasia, defect of articulation—all may appear as transient features in the pre-paralytic stage of general paralysis; and there may be sensory troubles of the same temporary character. There may be unilateral blindness or deafness, there may be neuralgias or numbness. More frequent is headache. Persistent severe headache, simulating the headache of cerebral tumour, occurs not very infrequently in the earliest stage of general paralysis.

The maladies with which general paralysis may be confounded are numerous. The melancholy form may be confounded with ordinary melancholy, the maniacal and exalted form with ordinary mania and non-paralytic exaltation; but in the ordinary malady the delusions never have the extreme hyperbolical exaggeration of general paralysis, and the affection of the pupils clears up the diagnosis at once when it is present, but it may have to be waited for, and it is not always safe to give a decision on a single interview. The main difficulty is, however, with the insanity arising from long continued alcoholic excess. Of course, there is the history, but even the patient's nearest friends—even his wife—does not always know that he is an habitual drunkard. A history of syphilis or its absence does not help us much, for alcoholics are not exempt from syphilis; and in 20 per cent. of general paralytics, as in 20 per cent. of other syphilitic maladies, there is no history of syphilis. Persistent morning vomiting does not occur except in pregnancy and chronic alcoholism, but it does not necessarily occur in the latter. Most important of all, the pupils in chronic alcoholism become sluggish in their reactions and it may be very unsafe to depend upon them for a diagnosis. The defect of memory is of a different type in the two diseases.

Fortunately two signs remain that are unimpeachable. The first is the result of lumbar puncture. If the cerebro-spinal fluid contain as many as half-a-dozen lymphocytes in the microscopic field of uncentrifugalised fluid, the case is certainly para-syphilitic; and with the presence of mental symptoms, general paralysis may be diagnosed with certainty. The other is the Wassermann reaction. If this is positive the malady is either general paralysis, tabes, or syphilitic meningitis. The presence of mental symptoms decides that it is general paralysis.

